# Turning Non-Sticking Surface into Sticky Surface: Correlation between Surface Topography and Contact Angle Hysteresis

**DOI:** 10.3390/ma17092006

**Published:** 2024-04-25

**Authors:** Jingyuan Bai, Xuejiao Wang, Meilin Zhang, Zhou Yang, Jin Zhang

**Affiliations:** 1 School of Intelligent Manufacturing, Lishui Vocational and Technical College, Lishui 323000, China; baekkyungwon@163.com; 2 School of Materials Science and Engineering, Northeastern University, Shenyang 110819, China; xuejiaowang0213@163.com (X.W.); zhangmeilin9696@163.com (M.Z.); 3 Engineering Research Center of Continuous Extrusion, Ministry of Education, Dalian Jiaotong University, Dalian 116028, China; yangzhou5959@163.com; 4 Key Laboratory of Near-Net Forming of Light Metals of Liaoning Province, Dalian Jiaotong University, Dalian 116028, China

**Keywords:** CuNi foam films, biaxially oriented polypropylene film, composite surface, contact angle hysteresis

## Abstract

We present a surface modification technique that turns CuNi foam films with a high contact angle and non-sticking property into a sticky surface. By decorating with mesh-like biaxially oriented polypropylene (BOPP) and adjusting the surface parameters, the surface exhibits water-retaining capability even when being held upside down. The wetting transition process of droplets falling on its surface were systematically studied using the finite element simulation method. It is found that the liquid filled the surface microstructure and curvy three-phase contact line. Moreover, we experimentally demonstrated that this surface can be further applied to capture underwater air bubbles.

## 1. Introduction

Surface topography has a great influence on wettability and, in particular, on the motion of droplets, with behaviors ranging from highly repellent to highly adhesive. Wetting-resistant surfaces, owing to their large water contact angle and non-sticking characteristics, have long been desired for a range of technological applications, including self-cleaning [1,2,3], anti-fogging [4], anti-icing [5], buoyance enhancement and drug reduction [6]. In terms of applications, nowadays in daily life and many industrial contexts, techniques such as spray [7], jet cooking [8], heat transfer [9] and/or microfluidics [10] require rather sticky surfaces. Recently, proper surface engineering approaches have become an important route to transfer liquid–surfaces adhesion. Decorating a flat surface with either hydrophobic or hydrophilic nanoparticles can create superhydrophilicity with high droplet adhesion [11]. Moreover, besides tailoring the adhesion properties, texturing the surface with chemical and physical heterogeneity also exerts a great influence on the in-air or underwater three-phase contact line. For example, the alternated superhydrophobic and hydrophilic strip surface produces a large contact angle hysteresis, preventing the movement of underwater bubbles.

The super-hydrophobicity of a surface is generally determined by two crucial factors: high surface roughness and low surface energy [12]. Three conventional approaches exist for the fabrication of foam films with superhydrophobic interfaces [13]: (a) Surface modification techniques can enhance the hydrophilicity of metal foam films by changing their surface properties and/or energy states. For example, plasma treatment [14], UV irradiation [15], and chemical modification-induced surface roughening [16]. (b) Designing the microstructure of metal foam films involves incorporating nano-level microstructures or microporous structures and using chemical methods to generate interfaces with desired morphology and chemical functionality in situ [17], thereby enhancing the surface area and roughness to improve hydrophilicity [13]. (c) To achieve the desired morphology, it is essential to implement appropriate surface modification techniques [18]. Additionally, coating the metal foam film with hydrophilic materials such as fluoropolymers or silanes [19] can effectively enhance its surface hydrophilicity. In comparison to alternative approaches, the application of a hydrophilic coating layer onto the metal foam film offers a simple, convenient, and cost-effective method. Moreover, by precisely controlling the material composition and thickness of the coating layer, it becomes feasible to tailor the hydrophilicity according to specific application requirements. However, it is crucial to acknowledge that coatings can be influenced by factors such as friction, aging, or chemical damage, necessitating regular maintenance and repair. Consequently, the selection of an appropriate coating material becomes important in ensuring the required performance and reliability in specific applications.

It has been demonstrated that this sticky phenomenon, also known as “petal effect”, is a consequence of significant contact pining, by which the droplets are affected in a complicated way by many surface topographies such as roughness factors and chemistries [20,21,22,23]. Pining of the contact line primarily prevents droplets from moving, and hence a sticky state describes systems with high contact angle hysteresis. In general, contact angle hysteresis is relevant to the tortuosity of the three-phase contact line and the maximum contact length scale. With proper surface engineering approaches, it is possible to manipulate the contact line structure including shape, length, continuity, and amount of contact. For example, microfabricated reentrant architecture creates a finger-like contact line, enabling highly wetting behavior for liquid/surface combinations that are typically non-wettable [24]. Rough surfaces can derive their low contact angle hysteresis from discontinuous contact [25]. The aforementioned studies have proved that manipulating surface topography should serve as a quick and efficient way for the design and engineering of surfaces with tailored adhesion properties.

There are few direct methods to observe the wetting state of a droplet on a microstructure surface, mainly limited to partial structures beneath the droplet. It is difficult to observe the internal wetting state within the droplet or to fully observe the entire wetting process of the droplet. Similarly, observing the wetting process of a droplet on a BOPP-CuNi metal composite film surface also presents challenges. In recent years, many studies have employed simulation methods to analyze the changes in the wetting state of droplets on surface microstructures. The most widely used numerical calculation methods are molecular dynamics simulation, lattice Boltzmann method, and computational fluid dynamics (CFD) simulation. These methods are, respectively suitable for microscale, mesoscale, and macroscale. Since the wetting process of real-sized droplets on specially structured surfaces involves macroscopic field changes, the computational fluid dynamics method (CFD) is more applicable for such studies [26,27,28,29,30,31].

The dynamics of the droplet impact is the main research area of CFD methods, with few studies focusing on the wetting state of droplets on microstructure surfaces. CFD methods are also commonly used in the field of microflow simulation, and the application of macroscopic-scale CFD methods to microscopic-scale research is a novel approach that can overcome the spatial and temporal limitations of molecular dynamics and lattice Boltzmann methods when calculating the wetting behavior of millimeter-scale droplets [32]. In this study, the CFD method was used to simulate the wetting process of droplets on a BOPP-CuNi metal composite film surface, investigating the specific wetting process of droplets on complex microstructure surfaces in a near-real size state. This study aims to address the shortcomings of existing research on the wetting process of liquids in near-real size and provide support for the “lotus effect” wetting transition theory. It is expected to provide empirical references for other related studies.

In this work, we propose a novel methodology of turning traditional non-sticking metallic foam film surface sticky by simply manipulating the surface parameter. The 3D CuNi foam films are prepared by one-step hydrogen bubble-assisted galvanostatic electrodeposition, while biaxially oriented polypropylene (BOPP) mesh with dissimilar surface roughness topologies, height and open area is used to decorate the Cu-Ni foam film surfaces. Recent developments in using the hydrogen template-assisted electrodeposition method have led to the synthesis of many foam films including Cu, CuNi, and Ni. These films typically contain macropores and, depending on the synthetic routes, nano porous dendritic walls, hence exhibiting a hierarchical porosity [33,34,35,36]. In this context, we demonstrated that the CuNi metallic foams, with a contact angle greater than 150, show non-stick properties to water droplets. In this study, composite surfaces configured of mesh-like BOPP and CuNi foam that constitute the “open area” are prepared. Contact angle and contact angle hysteresis were measured for each composite surface. It is also shown that the fraction of open area, and roughness of the BOPP can be adjusted to modify contact line pining and the adhesion properties. The correlation between these surface topographies and contact angle hysteresis is revealed.

## 2. Materials and Methods

### 2.1. Materials and Characterization

Scanning electron microscopy (SEM) was used to assess the surface morphology of CuNi foam films. Surface analyzer (LAUDA Scientific GmbH, Lauda-Königshofen, Germany, LSA-100) was used to determine the contact angles. The values reported are the averages of three measurements made on different areas of the sample surface. A 3D imaging software (VK-H1XAC, https://www.keyence.com.cn/) was used to measure the surface roughness. And each polished surface was measured two times at three different randomly selected locations.

### 2.2. Preparation of CuNi Foam Film

CuNi foams were electrodeposited from an electrolyte solution containing 0.5 M NiSO_4_·6H_2_O, 1.5 M H_2_SO_4_, 1 M HCl and 0.01 M CuSO_4_·5H_2_O. All electrodeposition experiments were carried out at room temperature in a 3-electrode cell connected to an electrochemical workstation (CHI760E). The working electrode was a pure copper plate with an active area of 1.5 cm^2^. The copper plate was well-polished with 1000, 2000, and 3000-grit SiC papers consecutively, and then washed with acetone, ethanol, diluted sulphuric acid and water. A platinum plate was used as a counter electrode. And a double junction Ag|AgCl 3 M KCl electrode (E = +0.210 V versus standard hydrogen electrode (SHE)) was utilized as the reference electrode. The CuNi foam films electrodeposition was performed galvanostatically at *j* = −0.67 A/cm^2^ and the deposition time was set at 100 s. Unless otherwise stated, all deposition processes were carried out at room temperature and under stirring (800 rpm) using a magnetic stirrer bar.

### 2.3. The Fabrication of Mash-like BOPP-Modified CuNi Foam Film Surface

**Figure 1:**
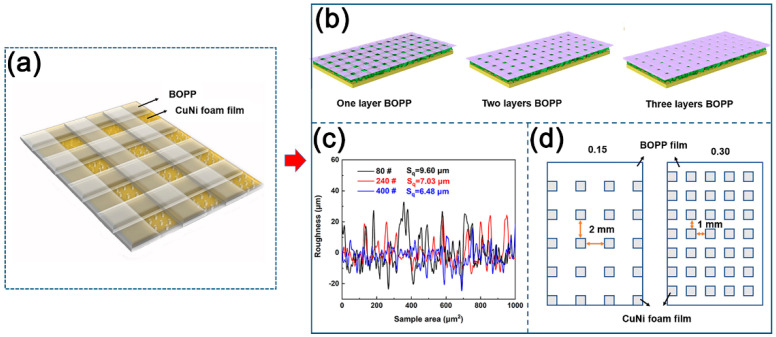
The schematic diagram of CuNi foam film and BOPP mesh strips composite surface (**a**). The yellow area is CuNi foam films area, and the purple-gray area is BOPP mesh strip area. The height of BOPP was adjusted by the number of layers of BOPP (**b**). Different roughness obtained by grinding with 80, 240, and 400-grit SiC sandpapers (**c**). The open area fraction between CuNi foam film and BOPP film (**d**).

Figure 1a depicts the images showing the BOPP mesh strips-decorated CuNi foam film surface. The width of the BOPP strips was set at 2, 1.5, and 1 mm, respectively, in order to adjust the area ratio of CuNi to BOPP. The height of the BOPP strips was adjusted by increasing the BOPP layers (Figure 1c). The strips were well-polished with 400-, 240-, and 80-grit SiC papers to produce different surface roughness. The prepared samples with different surface parameters are listed in Table 1.

### 2.4. FEM-Simulation of Wetting Dynamics

#### 2.4.1. Physical Model

**Figure 2:**
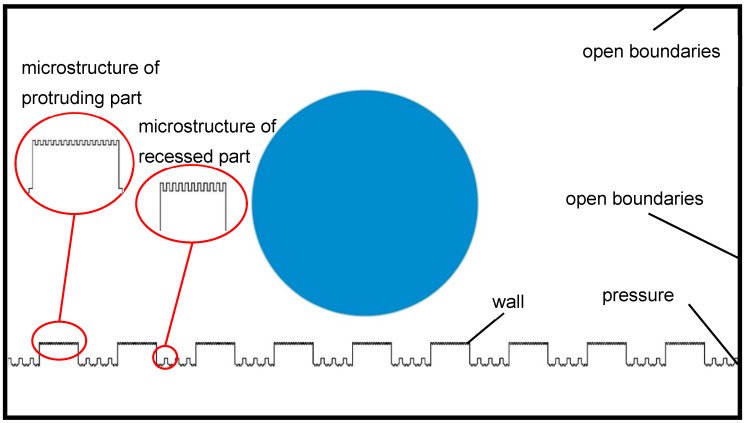
Geometric model of microstructure surface of CuNi foam films and BOPP.

Under the condition of being unaffected by external influences, the droplet possesses geometric characteristics of rotational symmetry [37]. Therefore, this study simplifies the problem by adopting a two-dimensional model to reduce computational complexity. The geometric model of the droplet wetting the mash-like BOPP-modified CuNi foam film surface is constructed using COMSOL Multiphysics simulation software (https://cn.comsol.com/), as shown in Figure 2. The computational domain is a rectangular region of 1 mm × 2.5 mm. The initial contact angle of the droplet on the CuNi foam film and BOPP surface is set based on the experimental results. The droplet diameter is set at 0.5 mm. The distance between the droplet and microstructure is set to be 60 mm, to prevent excessive mesh density caused by proximity and influence coming from impact phenomena. Since the droplet mass in the simulation model is small and it is in close proximity to the microstructure surface, it can be assumed that both the gas and liquid phases are in a laminar flow state. The physical properties of water and air in the two-phase flow are shown in Table 2 [38].

#### 2.4.2. Mathematical Model

This model considers the surface tension in the two-phase fluid flow process, and thus, the phase-field method is adopted, which includes more physical fields, for interface tracking. This method is advantageous for capturing subtle changes that occur during the droplet wetting process and achieving higher computational accuracy. In the simulation, water droplet is defined as the primary phase, and air as the secondary phase. The interface information is obtained through a defined phase-field variable (∅), eliminating the need to track the fluid interface changes through grid points. The conditions of the entire computational domain are set to closely approximate the real environment of contact angle measurements. It is defined as an isothermal flow field, where no heat loss occurs during the calculation, and the energy transfer does not need to be considered.

#### 2.4.3. Continuity Equation

Assuming that the viscous two-phase Newtonian fluids in the model are incompressible and immiscible, there exists an equivalent diffusive effect at the interface of this multiphase motion. The divergence of the velocity due to convection is zero, and the continuity equation is as follows:

(1) ∇·u=0

where ∇ represents the gradient operator, and · represents the dot product for vectors.

#### 2.4.4. Momentum Equation

As this model involves an incompressible laminar isothermal flow field, the introduction of the energy conservation equation is not necessary. Instead, the two-dimensional incompressible Navier–Stokes (N-S) equations, augmented with surface tension, are used to describe the system:

(2) $$\rho \left(\frac{\partial u}{\partial t}+\left(u\cdot \nabla \right)u\right)=-\nabla p+\nabla \cdot \left[\mu \left(\nabla u+\nabla u^{T}\right)\right]+F_{g}+F_{st}$$

where u is the velocity, ρ is the density, t is time, p is the pressure, μ is the dynamic viscosity coefficient, $F_{g}=\rho g$ represents the gravitational force, where g is the acceleration due to gravity, and $F_{st}$ is the volume force caused by the surface tension at the air/liquid interface.

#### 2.4.5. Initial and Boundary Conditions

To simulate conditions that are as close to the actual environment as possible, the top and sides are defined as open boundaries, while the microstructure is set as a wall boundary condition. The missing reference pressure point is the root cause of the non-convergence in solving the fluid flow problem. Defining a pressure point constraint at the lower right corner of the microstructure can avoid an uncertain pressure field and promote convergence in the solution process. The protruding surface of the microstructure is defined as wetting wall 1, while the recessed part is defined as wetting wall 2. Based on measurements from a contact angle measuring instrument, the contact angle of wetting wall 1 is set as hydrophilic, and the contact angle of wetting wall 2 is set as hydrophobic. The initial velocity of the droplet is set to 0, and the initial interfacial condition is a gas–liquid interface. The research method includes an initialization transient solution method. The total simulation time step is 0.2 s. To observe the wetting process of the droplet on the microstructure in detail and ensure computational accuracy while improving convergence, the computation time step is set to 0.00002 s.

#### 2.4.6. Finite Element Meshing

**Figure 3:**
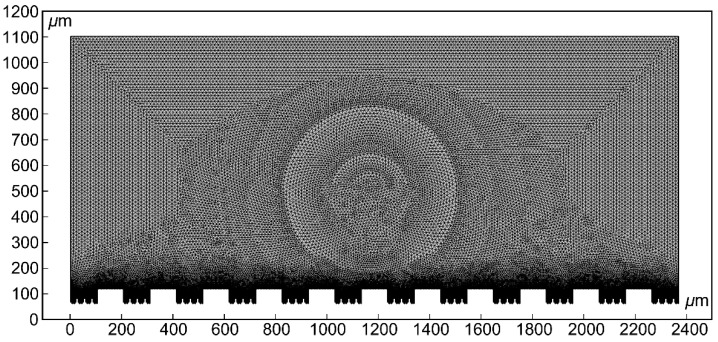
Mesh partitioning of geometric models.

The calculation results of numerical simulations are influenced by parameters such as grid size, type, and density. A higher grid density leads to more accurate simulation results, but it also increases computational requirements in terms of memory and time. For this purpose, a free triangular mesh is used for discretization, as it is more suitable for dividing the gas–liquid interface. The predefined grid size is highly refined, and the interface undergoes local grid refinement. The geometric scaling is set to 1. The resulting grid partition is shown in Figure 3.

## 3. Results and Discussion

### 3.1. CuNi Foam Film (before BOPP Modification)

**Figure 4:**
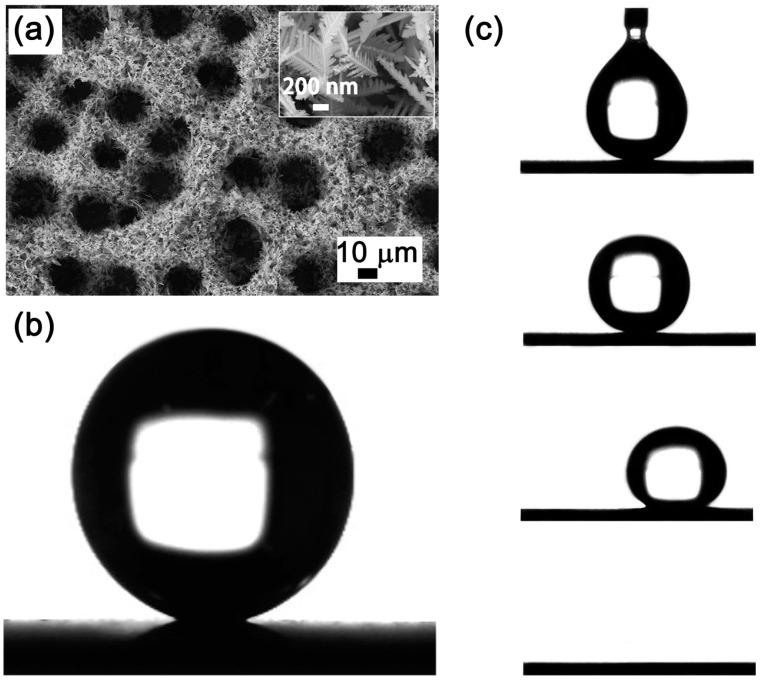
The microstructure (**a**) and static contact angle (**b**) of CuNi foam film. (**c**) Photographs of 10 μL water droplet rolling off the CuNi film surface titled by ~2°.

Experimental results elucidate that the interplay between contact angle and sliding angle is linked to surface morphology [39]. High-resolution SEM micrographs distinctly depict the as-fabricated CuNi foam films manifesting a three-dimensional, interconnected porous architecture. Within this structure, the dendritic walls present a hierarchical porosity, as illustrated in Figure 4a. Figure 4b delineates the wettability of 2 μL water droplets on the CuNi foam film surface, where the measured contact angle approaches 158°, signifying a pronounced Superhydrophobicity. It is imperative to underscore that the propensity for a droplet’s mobility on a substrate is governed more by the contact angle hysteresis than the contact angle alone [40]. Sequential images in Figure 4c elucidate the forces exerted by the syringe on the water droplet as it disengages from both the syringe and the CuNi film surface. Remarkably, the droplet neither permeates nor diffuses over the coating. Increasing the droplet’s volume to 10 μL leads to its detachment from the syringe due to gravitational forces. Yet, upon contact with the CuNi coating, it manifests a rolling behavior, and does not establish a permanent position on a CuNi foam film angled at 2°.

The randomness in pore positioning, resultant from the hydrogen bubble template during the electrodeposition process [41], hinders the establishment of a continuous solid–liquid contact line. This engenders the facile rolling-off of droplets upon minimal surface inclination. Such observations reinforce the conclusion that superhydrophilicity embodies not only high contact angle but also low contact angle hysteresis [42].

### 3.2. Mesh-like BOPP-Modified CuNi Foam Film Surface

**Figure 5:**
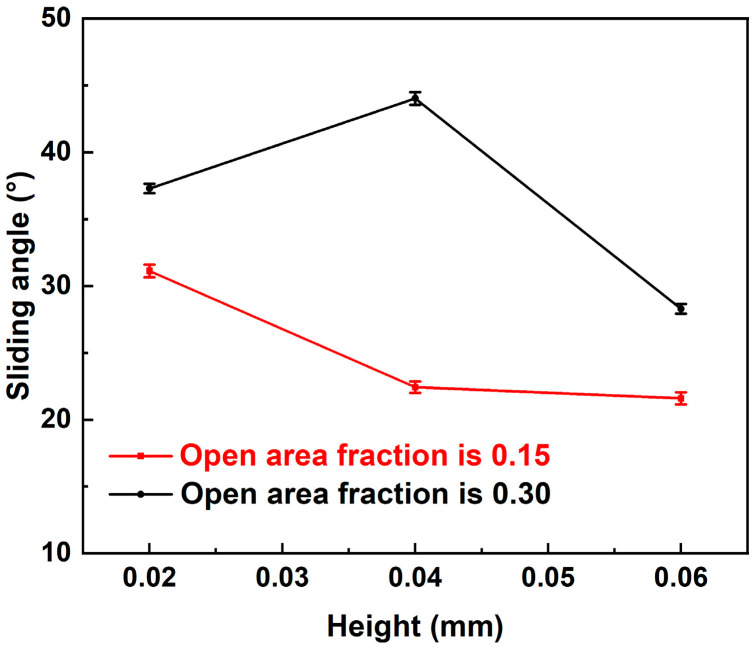
Experimental measurements of sliding angle vs. area fraction microstructures and BOPP films with different height.

**Figure 6:**
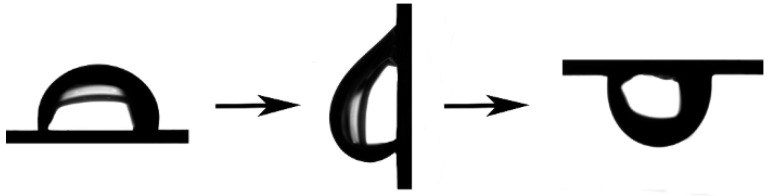
The contact angle of 30 μL droplet on surface of sample 7 at 0° tilt, 90° tile, and fully inverted, showing high droplet adhesion.

Quantitative measurements illustrated in Figure 5 exhibit the variation in sliding angles across the mesh-like BOPP-modified CuNi foam films differing in area fraction and height. Notably, for an area fraction of 0.30, the sliding angle first increases before diminishing with increasing BOPP height. Conversely, with an open area fraction of 0.15, the sliding angle gradually decreases while the height increases. In explicit terms, a surface having an area fraction of CuNi foam films and BOPP film of 0.3 and a height of 0.04 mm exemplifies optimal droplet adhesion, i.e., sample 5, whereas with an open area fraction of 0.15, a BOPP height of 0.02 mm realizes the most advantageous adhesion. Moreover, when the height is consistent, the adhesion of the open area fraction of 0.30 is greater than that of 0.15. But it has been experimentally verified, the graded roughness composed of the height and roughness values selected in this work does not exceed the allowed critical value of graded roughness. When the graded roughness is less than 14.2 µm, increasing the roughness can increase the contact angle hysteresis and increase the wetting step jump, improving the adhesion of droplets on solid surfaces [43].Therefore, choosing sample 7 to represent the roughness change of the BOPP surface, the BOPP was further polished with 240# sandpaper, and the corresponding images of the droplet on its surface when it is horizontal, vertical, and upside-down are shown in Figure 6. It is seen that the roughness of BOPP results in pronounced contact angle hysteresis, wherein the droplet remains steadfast, even upon inverting the composite surface.

The contact angle hysteresis intricately intertwines with area fraction, geometric characteristics, and spatial distribution [44]. In terms of the open area fraction, a more robust metric, the three-phase line ratio, emerges as a suitable descriptor for contact angle hysteresis. Within a model framework of square pillar distribution, the relationship between open area fraction and the three-phase line ratio is articulated as follows:

g = √f (3)

where ‘g’ represents the three-phase line ratio and ‘f’ symbolizes the open area fraction [45]. Experimental measurements suggest that an elevated three-phase line ratio increases the hysteresis, consistent with results derived from open area fraction evaluations. Therefore, a composite surface having an open area fraction of 0.30 emerges as the most propitious to engender adhesion to droplets. Conversely, the influence of BOPP’s height on the surface contact angle hysteresis does not exhibit a consistent pattern. Based on solid–liquid contact line theory, a droplet exhibits steadfastness on the surface when the pinning force at the contact line counterbalances the body force (attributable to the droplet’s weight) [46].

### 3.3. Fem-Simulation of Wetting Dynamics

**Figure 7:**
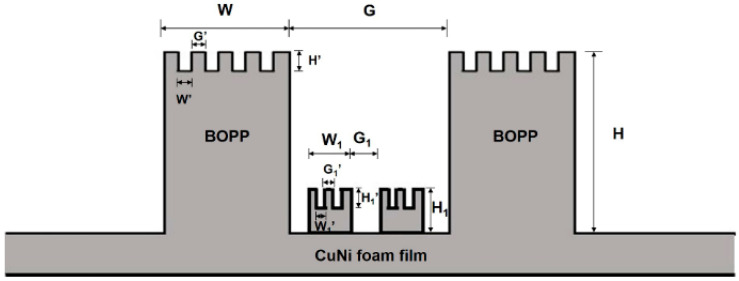
Model surface consisting of CuNi foam films and BOPP. For BOPP, the macroscale grooves are defined by groove width G, step width W, and step height H. Microscale grooves constitute macroscale grooves, which are defined by groove width G’, step width W’ and groove depth H’. The macroscale groove of the corresponding CuNi foam film is defined by groove width G1, step width W1 and step height H1 and microscale grooves are defined by groove width G1’, step width W1’ and groove depth H1’.

**Figure 8:**
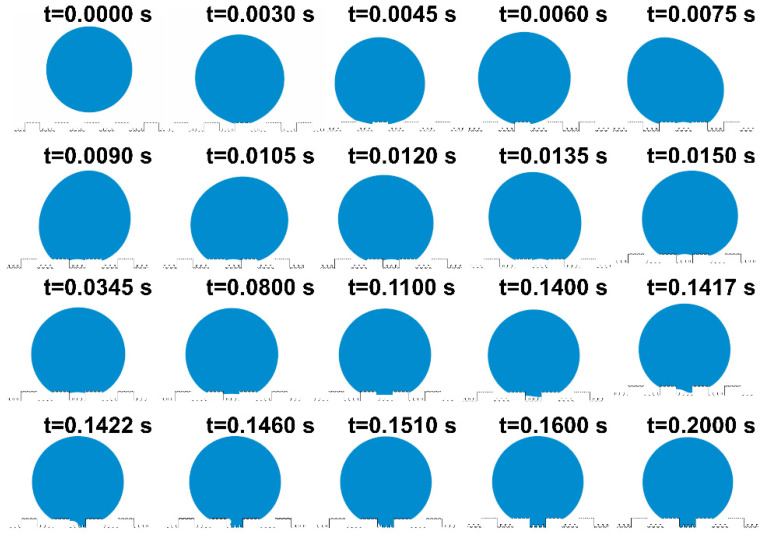
The process of droplets dropping and wetting microstructures.

To offer a succinct analysis, water droplets on the mash-like BOPP modified CuNi surface have been strategically simplified, effectively rendering the problem quasi two-dimensional. As shown in Figure 7, CuNi foam films manifest dual roughness dimensions: one at a nanometer scale offered by the dendritic wall—articulated in terms of groove width G1’, step width W1’, and groove depth H1’—and another at a microscale, typified by a three-dimensional porous configuration, denoted by groove width G1, step width W1, and step height H1. Polished BOPP exhibits bi-dimensional roughness: the micron-scale roughness post-polishing with 240# sandpapers is defined by groove width G’, step width W’, and groove depth H’; another dimension is the millimeter-scale roughness, defined by groove width G, step width W, and step height H. Based on this model, Figure 8 shows a series of dynamic images illustrating the droplet deposition process across distinct temporal intervals, as calculated through the phase field method. As the droplet descends, it engages with the microstructured surface, wherein the blue zone signifies the liquid phase, and the white represents the gaseous phase. Through computational simulations, the intricate interplay between the droplet and the microstructures becomes discernible. The entire wetting process embodies a series of intricate wetting states.

**Figure 9:**
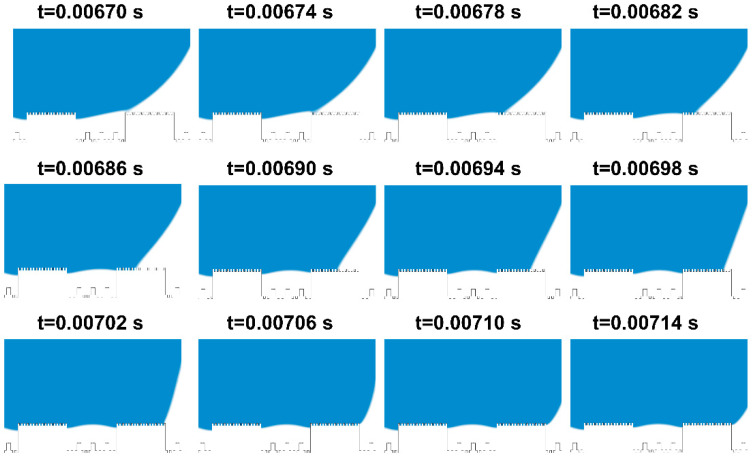
Enlarged view of droplet wetting process.

Initially, at t = 0 s, the droplet epitomizes a quintessential spherical contour. Subsequently, governed by gravitational forces, the droplet begins to move, overcoming its initial state of inertia. At t = 0.003 s, the bottom of the droplet comes into contact with the bulge and wets the protruding microstructure surface, resulting in the Wenzel wetting state. Above the three-phase contact line, the region is wetted, while below, it remains non-wetted. By this juncture, the droplet has fully wetted the protruding microstructure, exhibiting a slight rebound at t = 0.006 s. The enlarged view of the following states is shown in Figure 9. Starting from t = 0.0067 s, the droplet progressively wets the surface of each protruding microstructure until its manifestation as the Wenzel wetting state at t = 0.00714 s. During this interval, the droplet is subjected to the cumulative forces of surface tension, inertia, and pinning dynamics, inducing a translocation of its center of mass and noteworthy deformation. The droplet, in this scenario, is rendered incapable of sustaining its pristine spherical interface, undergoing temporal oscillations [47,48,49].

Figure 8 reveals that, following these oscillations, the droplet enters a transient equilibrium state at t = 0.0345 s. Unwetted regions within the microstructures trap some gas, exhibiting the Cassie state. A brief equilibrium wetting state appears at t = 0.1460 s. Under the influence of oscillations and gravity, the droplet progressively wets the recessed microstructures. When the droplet contacts a small cylindrical protrusion, it quickly makes contact with the bottom surface of the microstructure, and sequentially wets the remaining small cylinders and the entire grooves. This causes the gas to escape, eventually leading to a fully wetted stable state, where the three-phase contact line is anchored at the bottom of the microstructure.

From the state at t = 0.1417 s shown in Figure 8, the droplet is seen to wet the small cylinders of the recessed microstructure, rapidly moisten a portion of the substrate, and then penetrate the microstructures along its path. At t = 0.1600 s, the entire plane becomes wetted and stabilizes. The droplet stays pinned to the microstructure surface until the simulation concludes at t = 0.2000 s, without any subsequent changes.

Wetting phenomena on solid surfaces are conventionally articulated through the paradigms of “energy concept” and “pressure concept”. Specifically, the “energy concept” is particularly suitable for predicting non-uniform wetting states on rough surfaces, while the “pressure concept” is more suitable for analyzing the process of wetting transitions in hierarchical multiscale microstructures. It involves the expansion of the three-phase contact line and is based on the theory of pressure-induced wetting transitions. When the hydrostatic pressure within the liquid phase continuously increases and exceeds the breakthrough pressure, as defined by the following equation, the changes in the gas–liquid interface commence [50,51,52].

(4) $$p_{h}-p_{0}>p_{break}$$

where $p_{0}$ represents the air pressure at the bottom of the microstructure, $p_{h}$ represents the hydrostatic pressure, and $p_{break}$ represents the breakthrough pressure. When the pressure difference surpasses a critical value, the wetting transition initiates, and the contact line begins to expand.

In the wetting process of hierarchical multiscale microstructures, the pressure-induced wetting transition plays a crucial role. Upon the droplet’s engagement with the microstructures, the pressure within the droplet increases due to the confinement and compression of the liquid phase. Such a surge amplifies the wetting force, fostering a progressive dilation of the contact line to subsume the microstructural surfaces. The pressure-induced wetting transition can be further understood by considering the balance among the interfacial tension, the capillary pressure, and the pressure within the droplet. When the pressure within the droplet exceeds a certain threshold, the force balance undergoes perturbation, leading to the expansion of the three-phase contact line and the transition to a new wetting state.

Overall, the pressure concept is particularly relevant for analyzing the wetting transition process in hierarchical multiscale microstructures, where the pressure difference between the liquid and gas phases determines the occurrence of wetting transitions and the expansion of the three-phase contact line.

The wetting process of droplets on microstructures can be categorized by the dynamics of the three-phase contact line. There are two primary modes of breakthrough at the gas–liquid interface, each triggered by a critical pressure difference. The first mode, known as the Canthotaxis effect, involves the three-phase contact line advancing along the surface, with the gas–liquid interface descending. In the second mode, called the Laplace breakthrough, the three-phase contact line remains anchored at sharp geometric edges while the gas–liquid interface expands.

**Figure 10:**
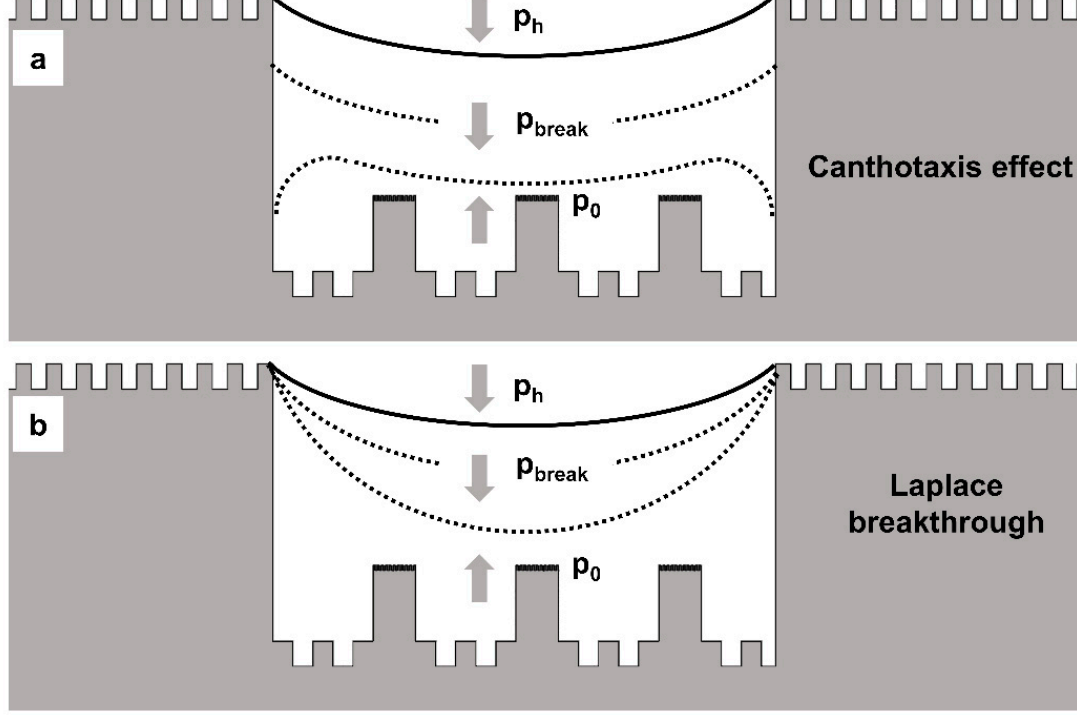
Patterns of droplet wetting of hierarchical multi scale microstructures. The breakthrough pressure in the Canthotaxis effect mode is given by the following equations. (**a**) Canthotaxis effect. (**b**) Laplace breakthrough.

Figure 10 illustrates the alterations in the gas–liquid interface caused by varying pressures in these two scenarios. As per the Laplace law, the curvature radius of the gas–liquid interface is dictated by the pressure difference and the inherent contact angle of the interface. When the three-phase contact line is anchored to protrusions on the microstructure, a continuous increase in the droplet’s hydrostatic pressure results in the gas–liquid interface intruding into the recessed microstructure. Based on the formula for curvature radius, assuming that the intrusion of the gas–liquid interface does not result in compression of the air inside the microstructure, the pressure in this region does not increase. As a result, the gas–liquid interface forms a cap-like shape.

(5) $$p_{C,break}=\frac{4\sigma sin\theta_{i}}{S}$$

In the Laplace breakthrough mode, the expression for the breakthrough pressure is the following:

(6) $$p_{L,break}=\frac{4\sigma }{S}$$

where *S* denotes the spacing between microstructures, *σ* represents the surface tension of the droplet, and $\theta_{i}$ is the intrinsic contact angle between the solid and liquid.

From the breakthrough equation, it is evident that $p_{L,break}$ is significantly greater than $p_{C,break}$. In this model, the wetting process of the droplet is predominantly governed by the intrinsic contact angle. The surface of the microstructure’s protrusions has an intrinsic contact angle that is hydrophilic. Therefore, when the droplet wets the edges of the microstructure, it results in the pinning effect of the three-phase contact line. This pinning continues until the gas–liquid interface reaches the convex pillar situated in the recessed region of the microstructure, signaling the termination of the pinning effect. Consequently, the droplet, in a near-steady state, briefly undergoes wetting in the $p_{L}$ mode before swiftly wetting the entire microstructure in the $p_{C}$ mode.

**Figure 11:**
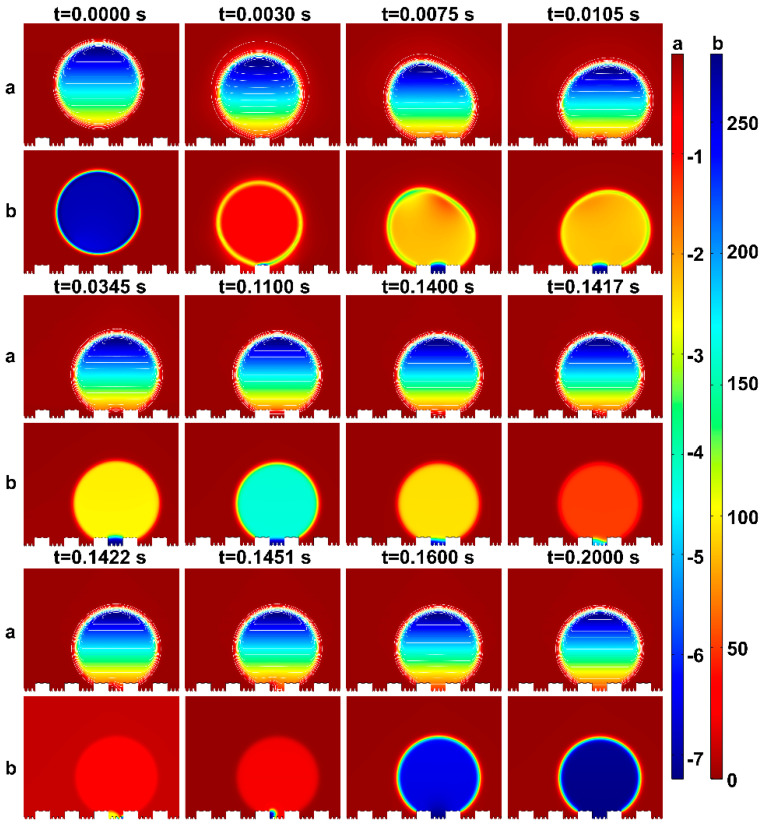
Hydrostatic pressure and pressure distribution diagram of droplet wetting microstructure process: a is hydrostatic pressure; b is pressure.

The graph depicted in Figure 11 presents both the static water pressure and the pressure variations as the droplet wets the microstructure. It can be observed that the static water pressure of the droplet incrementally rises from bottom to top which is attributable to gravitational effects. The pressure on the droplet’s outer surface is lower than the internal pressure distribution, which is an outcome of surface tension forces. This observation aligns with the insights provided by the pressure distribution graph.

When the static water pressure within the droplet surpasses the air pressure confined at the base of the microstructure, and their difference exceeds the breakthrough pressure, the three-phase contact line begins to shift. In this scenario, however, the gas phase trapped in the recessed part of the microstructure is compressed by the droplet, leading to an elevation in air pressure. Consequently, as the three-phase contact line expands, the gas–liquid interface does not assume the cap-shaped form discussed in the two modes. Instead, it undergoes an initial uniform wetting of the recessed morphology, achieving a temporary equilibrium state, before swiftly transitioning to non-uniform wetting. This behavior can be attributed to the trapped air, which acts as a pressure buffer opposing consistent wetting. In the pressure-induced wetting transition process, characterized by a hydrophilic intrinsic contact angle, capillary forces drive the initial downward infiltration of the droplet into the cavity, creating a concave fluid interface. As the static water pressure keeps rising, the three-phase contact line descends along the sidewall. A transient equilibrium flat-interface morphology occurs when counterbalanced by the confined air, which is subsequently followed by non-uniform wetting.

In summary, during the quasi-steady state, the droplet operates in the Laplace breakthrough mode. However, as it stabilizes, the droplet transitions from the uniform wetting state, undergoing a wetting shift, and ultimately aligns with the Canthotaxis effect mode. This theoretical analysis and the computational results of the transition from the Cassie state to the Wenzel state are consistent with one another. This congruence offers an alternative explanation for the pronounced contact angle hysteresis exhibited by droplets on hierarchical multiscale microstructure surfaces.

**Figure 12:**
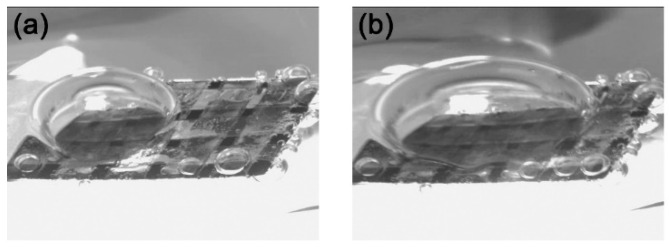
Stability of bubbles on the surface of sample 7: (**a**) 200 r/min; (**b**) 500 r/min.

Similarly, the fabricated surface demonstrates a capacity to seal an air layer underwater to some extent. Figure 12 shows the ability of sample 7 to retain air bubbles when submerged in water. The camera’s perspective is aligned with the direction in which bubbles adhere to the surface. When bubbles are introduced onto this surface and agitated by a magnetic stirrer, they remain stable on the surface, even when disrupted at rotations of 200 r/min and 500 r/min. This demonstrates that the fabricated surface provides adhesion not only for water droplets but also for air bubbles.

## 4. Conclusions

In this study, surfaces with high droplet adhesion were created by decorating CuNi foam film with mesh-like BOPP. The three-phase contact line ratio is used to judge the influence of the open area fraction on the contact angle hysteresis of the composite surface. A large three-phase contact line ratio leads to a large contact angle hysteresis and good adhesion. The three-phase contact angle ratio selected in this letter is √0.30, exhibiting the highest adhesion. The multiscale hierarchical composite roughness composed of BOPP and CuNi foam film with a different roughness and height can make the contact angle hysteresis of droplets on it greater than 90°, even if it reaches 180°. The sources and wetting process of wetting resistance were accurately described through finite element numerical calculations, further elucidating the mechanism of wetting transition in hierarchical porous CuNi composite films. In addition, the prepared surface can also realize the adhesion of air bubbles underwater.

## Figures and Tables

**Table 1:** The prepared patterned hydrophilic–hydrophobic heterogeneous surfaces and the corresponding parameters.

Sample	Area Ratio of CuNi to BOPP	Height of BOPP (μm)	Sq Roughness of BOPP (μm)
1	0.15	20	un-treated
2	0.15	40	un-treated
3	0.15	60	un-treated
4	0.3	20	un-treated
5	0.3	40	un-treated
6	0.3	60	un-treated
7	0.3	40	9.607

**Table 2:** Physical parameters of water droplet and air.

	Density (kg/m^3^)	Surface Tension (N/m)	Dynamic Viscosity (Pa·s)
Water droplet	998.2	0.0728	0.001003
Air	1.225	/	0.0000179

## Data Availability

Data are contained within the article.
